# Sexual Dimorphism of the Zebra Finch Syrinx Indicates Adaptation for High Fundamental Frequencies in Males

**DOI:** 10.1371/journal.pone.0011368

**Published:** 2010-06-29

**Authors:** Tobias Riede, John H. Fisher, Franz Goller

**Affiliations:** 1 Department of Biology, University of Utah, Salt Lake City, Utah, United States of America; 2 National Center for Voice and Speech, University of Utah, Salt Lake City, Utah, United States of America; 3 Department of Physiology, University of Utah, Salt Lake City, Utah, United States of America; University of Auckland, New Zealand

## Abstract

**Background:**

In many songbirds the larger vocal repertoire of males is associated with sexual dimorphism of the vocal control centers and muscles of the vocal organ, the syrinx. However, it is largely unknown how these differences are translated into different acoustic behavior.

**Methodology/Principal Findings:**

Here we show that the sound generating structures of the syrinx, the labia and the associated cartilaginous framework, also display sexual dimorphism. One of the bronchial half rings that position and tense the labia is larger in males, and the size and shape of the labia differ between males and females. The functional consequences of these differences were explored by denervating syringeal muscles. After denervation, both sexes produced equally low fundamental frequencies, but the driving pressure generally increased and was higher in males. Denervation strongly affected the relationship between driving pressure and fundamental frequency.

**Conclusions/Significance:**

The syringeal modifications in the male syrinx, in concert with dimorphisms in neural control and muscle mass, are most likely the foundation for the potential to generate an enhanced frequency range. Sexually dimorphic vocal behavior therefore arises from finely tuned modifications at every level of the motor cascade. This sexual dimorphism in frequency control illustrates a significant evolutionary step towards increased vocal complexity in birds.

## Introduction

In many animals, vocal behavior plays an important role in reproduction in both male-male and male-female interactions. Vocal behavior is therefore often sexually dimorphic. In songbirds, the vocal repertoire of males is typically larger than that of females [1], and the most complex vocalization, song, develops through vocal learning [2]. In some species, as for example the zebra finch (*Taeniopygia guttata*), only the male sings [3].

Vocal behavior in songbirds is generated through sophisticated neural control [4], which must integrate movements of multiple peripheral motor systems controlling respiration [5], the vocal organ [6], [7] and upper vocal tract structures [8]. Sound production occurs in highly specialized organs of the airways, which consist of a cartilaginous framework, oscillatory soft tissues, and muscles for airflow and vocal control [9].

To what degree sexually dimorphic vocal behavior arises from differences in central neural control and differences in the functional morphology of peripheral organs is an important question for understanding the evolution of vocal behavior. In the zebra finch, male song and male distance calls are learned vocal patterns, which are characterized by a diverse array of acoustic components, ranging from low-frequency sounds (480–1200 Hz) with rich upper harmonic content to more tonal high-frequency sounds (3–7 kHz). Females do not sing, and the female distance call is not learned. It contains only low frequency components and less pronounced frequency modulation than that of males. These behavioral differences are paralleled by differences in the neural architecture and biochemistry of the song control pathways, which give rise to learned vocal behavior in males (distance calls and song) but not in females [10], [11], [12].

It is still unclear to what degree this sexual dimorphism in central vocal control is also accompanied by dimorphic functional morphology of the sound generating organ. The male syrinx is controlled by a substantially larger muscle mass than that of females [13], [14], [15], [16], [17], and these muscles can contract at higher rates [18]. However, it is not known whether these differences are manifested in a simple increase in force, thus explaining a wider fundamental frequency range, or the potential for more rapid temporal control of vocal parameters. If these differences in muscle mass and contractile properties were translated into larger biomechanical differences through modifications of morphology and histology of the sound generating apparatus, an amplification of neural and morphological changes into acoustic potential would occur. Whereas for the songbird syrinx the intraspecific variation in syringeal morphology and histology has not been investigated, such differences have been established in mammals where they give rise to pronounced sexual dimorphism in vocal characteristics. For example, the sex-specific frequency difference in the human voice is based on larynx size [19] and on biomechanical properties of the vocal fold tissue [20], [21].

Here we investigate in the zebra finch whether morphology and histology of the sound generating structures differ between males and females. We explore the functional morphology of sound generation and show that differences in the vibrating tissues and in the cartilaginous framework are consistent with the production of a greater range of sound frequencies in males than females. These results therefore strongly suggest that sexually dimorphic vocal behavior arises from modifications at every level of the complex sound production system. Small modifications at the peripheral organ parallel those of the muscular and the neural control systems to give rise to a substantial increase in acoustic diversity.

## Results

### Histology and morphometry

**Figure 1:**
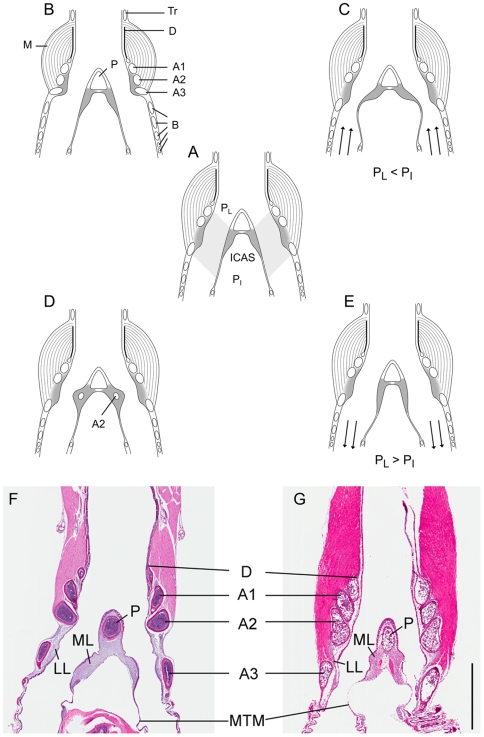
Schematic and H&E stained frontal sections through the syrinx. **A–E** illustrate active, muscular and passive, pressure-driven control of the syringeal valve (corresponding to “active and passive closure models” of Gaunt, [22]). Stained sections of a female (**F**) and male (**G**) syrinx at about mid organ level illustrate sexual dimorphism in muscle mass and bronchial half rings. Syringeal muscles adjust the position of the labia and therefore adjust the valve from complete closure to active opening. A partially adducted position is also assumed for induction of phonation [23]. **A.** Neutral position of labia. **B.** Rotation of the third bronchial half ring moves the lateral labium into the bronchial lumen [24], [25]. **C.** The first and second bronchial half rings arch with their end points into the ventral and the dorsal aspect of the medial labium and therefore tense the labial tissue but also exert control over its position [9]. The valve control mechanisms in **B** and **C** most likely occur simultaneously, but are effected by different syringeal muscles. The dorsal and ventral tracheobronchial muscles act as adductor and abductor of the lateral labium, respectively [26], [27], [28]. The role of the ventral syringeal muscle appears to include abductive activity, as indicated by its activation during the expiratory phase during quiet respiration [6], [29]. **D** and **E.** A second mechanism for adjusting labial position is passive. The syrinx is located inside the interclavicular air sac (ICAS). Pressure variation inside the subsyringeal air sac system causes similar pressure variation in the ICAS. The medial labium therefore passively moves into or out of the bronchial lumen if there is a pressure differential between the bronchial lumen (P_L_) and the ICAS (P_I_) (first described by Hérissant in 1753, after Gaunt [22]). ICAS pressure is larger than pressure in the bronchial lumen during expiration and smaller during inspiration. Muscle activity synchronized with the respiratory cycle [6], [29] keeps the syringeal lumen open during expiration. Without muscle activity the lumen closes during expiration (**D**) and opens during inspiration (**E**). During expiration, the medial and lateral labia are sufficiently adducted that increased flow causes self-sustained oscillations of the labia and, thus, generates sound. Phonation is maintained as long as an asymmetric shape of the labia is combined with flow separation right behind the labia as long as the driving pressure is high enough [23]. Abbreviations: P, pessulus; A1, A2, A3, three bronchial half rings; ML, Medial labium, MTM, Medial tympaniform membrane, LL, Lateral labium, Tr, tracheal ring; D, drum; M, intrinsic syringeal muscles; B, bronchial ring; arrows in **C** and **E** indicate air flow direction. The bar in **G** indicates a 1 mm distance and applied to **F** and **G**.

The histological composition of the labia (supplemental information S1 and supplemental figures S1, S2, S3, S4, S5) as well as the dorso-ventral and left-right variation did not differ between males and females (supplemental information S1 and supplemental figure S6). However, the size and shape of some syringeal components was sexually dimorphic (Figure 1) (Table 1). The left and right first bronchial half rings (A1) were larger in males (Table 1). Measurements for the right second half ring (A2-r) approached significance (t = −1.96; P = 0.07). The left lateral labium (LL) was larger in females (Table 1). The differences in the length of the left medial labium (ML) approached statistical significance (t = −2.14; p = 0.057). The left medial tympaniform membrane (MTM) is significantly longer in females (Table 1). Because combined length of the left ML and MTM was not different between males and females (Table 1), and the area of the left ML is also similar (Table 1), the shape of the left ML but not its size was sexually dimorphic. There was no lateral asymmetry between the left and right medial aspect of the syrinx (combined ML and MTM length) in males (paired t-test, N = 6, t = 0.06, P = 0.95) or females (paired t-test, N = 6, t = −1.20, P = 0.28). The measurements for other parameters (P, A2-l, A3-l, A3-r, ML-area) were not found to be sexually dimorphic.

**Table 1:** Average data, coefficients of variation (CV) and t-tests comparing males and females for 18 measurements.

Parameter	length in µm; area in µm^2^; mass in g; N_m,f_ = 6	CV (%)	significance	male-female ratio
P(area)	m: 51038±9353f: 51035±8668	18.316.9	t = −0.0006P = 0.99	1.00
A1-l(area)	m: 81206±16762f: 46223±6152	20.613.3	t = −4.79P<0.001 **	1.75
A2-l(area)	m: 83140±15936f: 83670±9045	19.210.8	t = −0.07P = 0.94	0.99
A3-l(area)	m: 63927±20659f: 62949±14523	32.323.1	t = −0.09P = 0.92	1.01
A1-r(area)	m: 76623±18411f: 56040±8092	24.014.4	t = −2.5P<0.05 **	1.36
A2-r(area)	m: 89647±16610f: 73871±10471	18.514.2	t = −1.96P = 0.07	1.21
A3-r(area)	m: 67434±24756f: 59322±16642	36.728.0	t = −0.66P = 0.51	1.13
LL-area-l	m: 67938±24332f: 106828±25195	35.823.6	t = 2.7P<0.05 **	0.63
ML-area-l	m: 95864±36517f: 85576±37784	38.144.1	t = −0.47P = 0.64	1.12
ML-length-l	m: 949±201f: 718±167	21.223.3	t = −2.14P = 0.057	1.31
MTM-length-l	m: 446±95f: 642±134	21.220.8	t = 2.92P<0.05 **	0.69
combined ML-MTM length-l	m: 1395±287f: 1361±121	20.68.9	t = −0.26P = 0.79	1.02
LL-area-r	m: 59612±17471f: 98526±40612	29.341.2	t = 2.15P = 0.056	0.60
ML-area-r	m: 92982±29263f: 88182±37399	31.542.4	t = −0.24P = 0.81	1.05
ML-length-r	m: 939±231f: 777±220	24.628.4	t = −1.24P = 0.24	1.20
MTM-length-r	m: 460±131f: 521±90	28.417.3	t = 0.94P = 0.37	0.88
combined ML-MTM length-r	m: 1399±284f: 1297±211	20.316.3	t = −0.69P = 0.50	1.08
Body mass	m: 13.7±0.4f: 11.8±0.7	2.86.2	t = −5.67P<0.001	1.16

### Bilateral neurotomy

**Figure 2:**
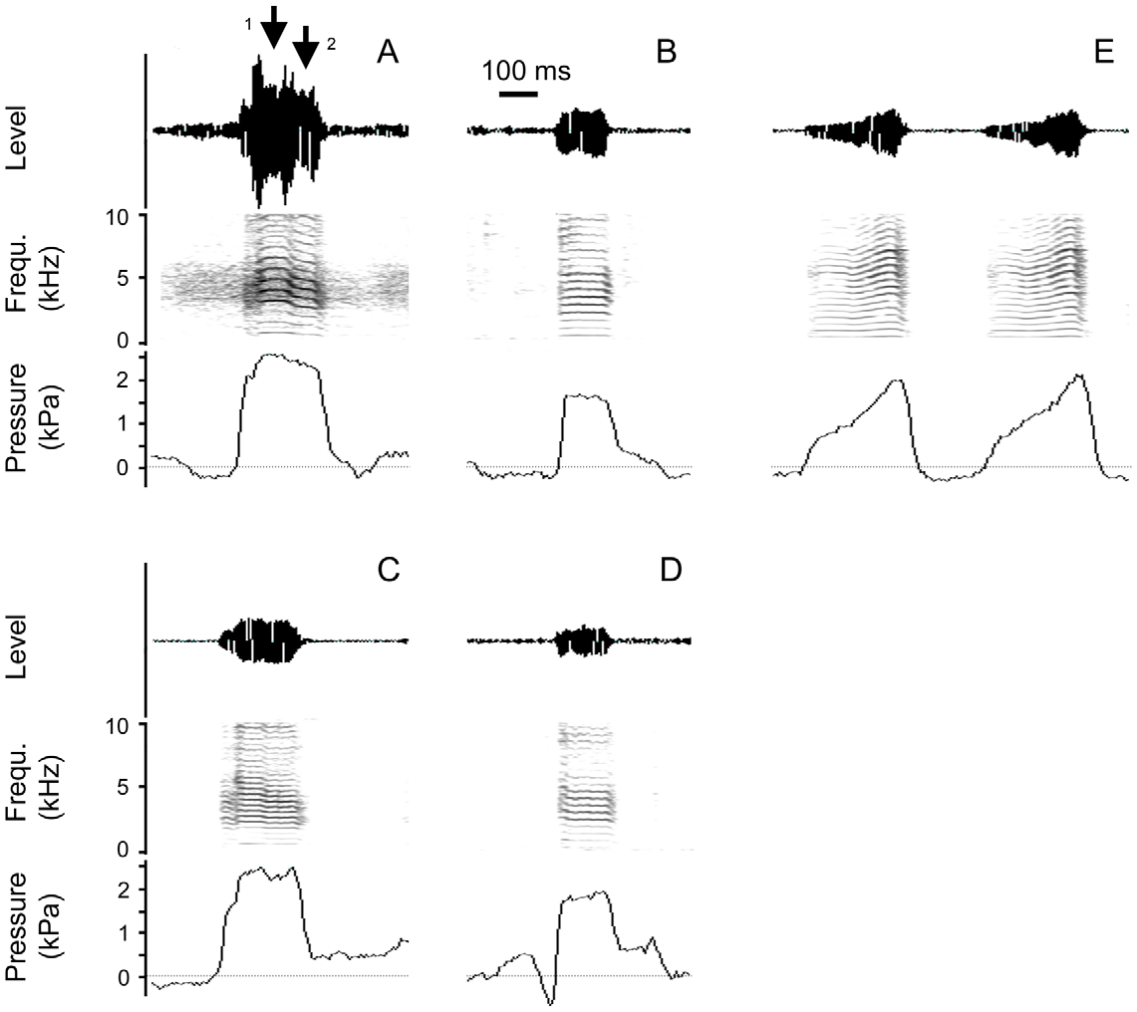
Oscillogram (top panel), spectrogram (middle panel) and subsyringeal air sac pressure (bottom panel) of a male (A, before nerve cut, C, same individual after nervecut) and a female (B, before nerve cut, D, same individual after nervecut) distance call as well as of two representative examples of respiratory sounds (E). The two arrows in **A** indicate a high (1) fundamental frequency at the beginning of the male call and a low (2) fundamental frequency in the middle and end of a male distance call. This frequency modulation does not occur in females and was also missing in one of the three males in this study. Calls in **A** and **B** are representative before nervecut surgery, and **C** and **D** are examples from the same individuals after tracheosyringeal nervecut.

**Figure 3:**
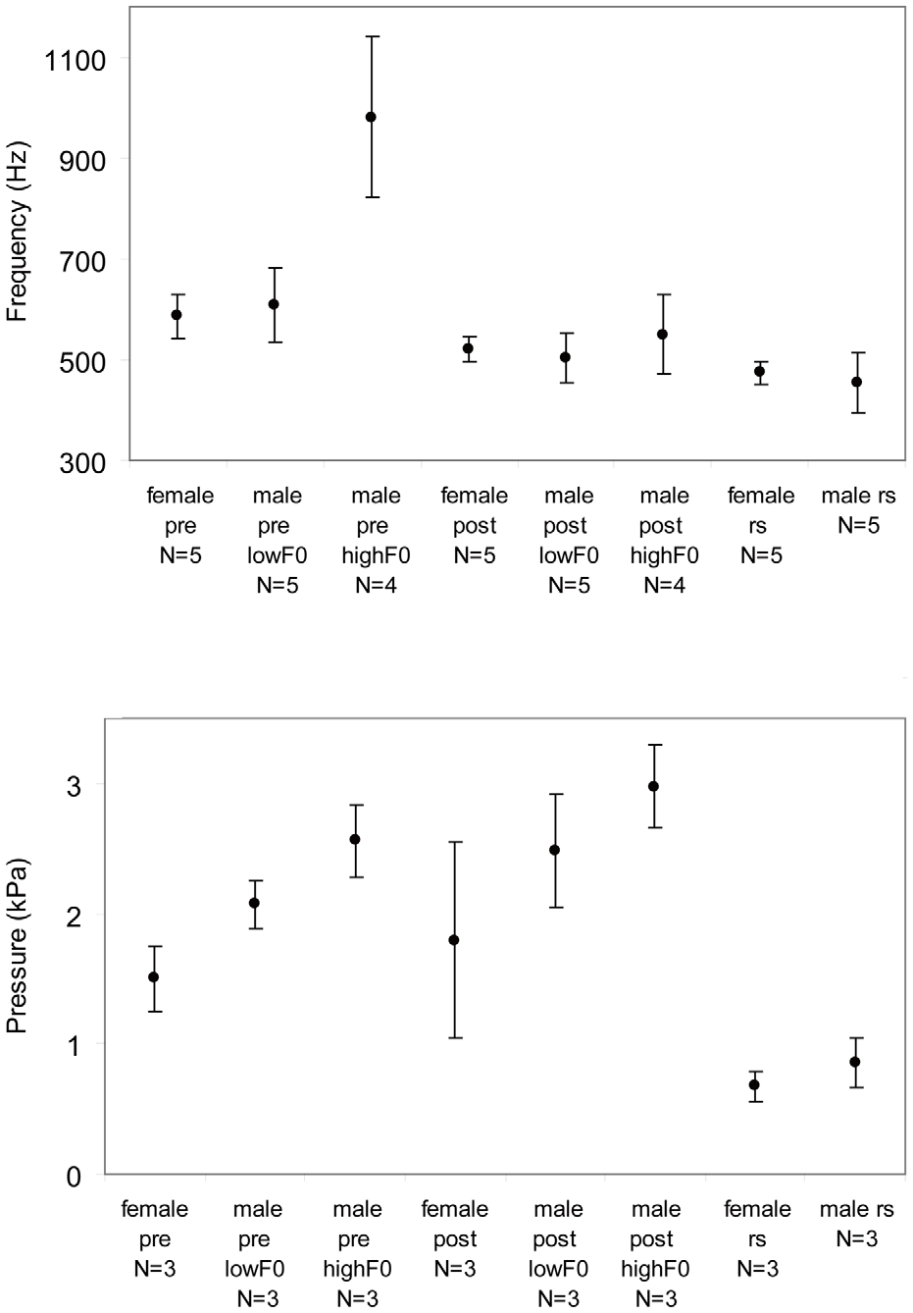
Fundamental frequency and air sac pressure measurements (mean ±1 standard deviation) in female and male calls before (“pre”) and after bilateral neurotomy (“post”). Fundamental frequency and air sac pressure was also measured in the respiratory sounds (“rs”). ‘High’ and ‘Low’ fundamental frequency (F0) in male calls (“highF0” and “lowF0”) is explained in Figure 2. Means were calculated from individual means (number of individuals, N, indicated in each category) which are based on measurements in twenty calls. The probability levels that the slopes are greater than zero is P = 0.006 before and P = 0.012 after the nervecut.

Acoustic parameters for male and female distance calls were measured (Figure 2). Average fundamental frequency in female distance calls ranged from 534 to 652 Hz. The low frequency component of male distance calls ranged from 504 to 682 Hz (Figure 3) and is not significantly different from the frequency of female calls (N_1,2_ = 5, ANOVA, F = 0.6, P = 0.46). In addition, male calls contain a high-frequency component (range 810 to 1157 Hz) that is absent in female calls.

In all individuals, fundamental frequency of distance calls was significantly lower after bilateral neurotomy (Figure 3). Mean fundamental frequency in female calls ranged from 506 to 559 Hz. The high fundamental frequency in male calls dropped to values between 446 and 627 Hz, while the low frequency component ranged from 427 to 546 Hz. The difference between the low frequency component of male calls and female calls was not significant (N_1,2_ = 5, ANOVA, F = 0.56, P = 0.47).

The respiratory sounds showed a rich harmonic structure and were produced with subsyringeal air sac pressure pulses that showed steadily increasing pressure throughout the expiration (Figure 2). This modulation in pressure was accompanied by an increase in fundamental frequency of the respiratory sound. The fundamental frequency at the onset of the respiratory sounds is therefore the lowest measured frequency for each individual. The lowest fundamental frequency in the respiratory sounds was not significantly different in males and females (N_1,2_ = 5, ANOVA, F = 0.5, P = 0.49).

**Figure 4:**
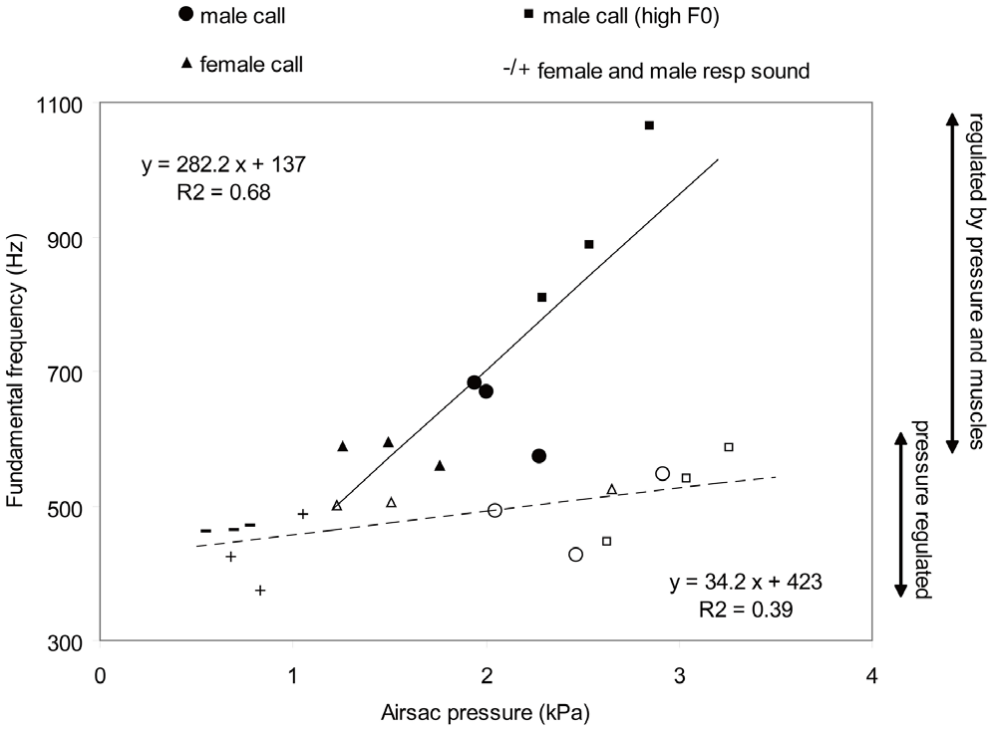
Relationship between fundamental frequency and subsyringeal air sac pressure. Before nervecut (full symbols); after nervecut (open symbols). Respiratory sounds in males (+) and females (-) are only present after the nervecut. The regression lines were calculated using all pre-neurotomy data (solid line) and all post-neurotomy data (dashed line). The arrows on the side indicate fundamental frequency ranges which are regulated by abdominal pressure or by a combination of abdominal pressure and muscle activity. Pressure and muscle regulated range extends up to 4 kHz for some song syllables. Pressure in those high frequency song syllables does not exceed 5 kPa (data not presented here).

Fundamental frequency is determined by size and tension of the labia, but also by subsyringeal air sac pressure. Variation in subsyringeal pressure can partially explain variations in fundamental frequency, as the positive regression between fundamental frequency and pressure before and after the neurotomy suggests (Figure 4).

In 3 males and 1 female, the subsyringeal pressure driving the production of distance calls increased after the neurotomy (Figure 3B). Most interesting for this study is the question whether the different fundamental frequencies in the respiratory sounds between males and females can be explained by pressure differences. One would expect females to generate the slightly higher frequency with higher pressure, if their sounds were produced with an identically designed sound source. However, this prediction is not supported by the results. Subsyringeal air sac pressure during the respiratory sounds was not significantly different in males and females (N_1,2_ = 3, ANOVA, F = 2.0, P = 0.22) (Figure 3). Furthermore, males produced the low frequency part of their distance calls with slightly higher subsyringeal air sac pressure than females before (N_1,2_ = 3, ANOVA, F = 10.3, P<0.05), but not after the nerve cut (ANOVA, F = 1.86, P = 0.24).

The regression analyses of subsyringeal air sac pressure and fundamental frequency showed a dramatic change in the relationship between the two parameters after the neurotomy (Figure 4). The slope of the regression line for the intact syrinx is significantly larger (ANCOVA, F = 21.2; Df n = 1, d = 19; P<0.001) than the one for the denervated syrinx, indicating that pressure variations only caused very small variation in fundamental frequency compared to that present in the intact syrinx.

## Discussion

Here we demonstrate morphological characteristics of the female zebra finch syrinx as well as sexually dimorphic features of the cartilaginous framework and of the sound-generating labia in the syrinx of the zebra finch. Just like in males [9], morphological differences between the left and right side of the syrinx in female zebra finches were not consistent (Supplemental information). In this species, the lateralization of high-frequency sound production in males [6], [30], [31] and possible lateralization in females cannot be explained by a simple size difference between the two sound sources. In many songbird species each side of the syrinx is specialized for a specific frequency range [32]. In the absence of morphological asymmetry, an alternative mechanism for tuning sound sources to different frequency ranges could include differences in the viscoelastic properties of the labia of the left and right syrinx. Such a mechanism was implicated in accounting for frequency range differences in mammalian vocal folds [33].

Together with physiological evidence on frequency control in the intact and denervated syrinx, these morphological differences indicate that the male syrinx is specialized for production of high frequencies. This evidence, in combination with earlier findings on the sexually dimorphic syringeal musculature [13], [14], [15], [16], [17], [18], provides a biomechanical mechanism for transducing sexually dimorphic neural instructions into different acoustic behavior, thus illustrating a functional sexual dimorphism at the level of the sound generating organ. In the following we discuss the biomechanical effects of morphological differences in more detail and suggest an evolutionary step from more passive to more active frequency control that allows expansion of the frequency range of the sound source in birds.

The morphological difference in the shape of cartilage rings (both A1, and possibly the right A2) can be directly translated into control of fundamental frequency of sound. The medial labia are fixed between the endpoints of cartilages A1 and A2, while the lateral labia sit inside the inner arch of these half rings. Because sound frequency is modulated by regulating the tension of the labia, a simple bending of one or both half rings will stretch both labia. The combination of a muscle-generated outward bending of A1 and/or A2 and inward returning movement of the half ring by elastic recoil would allow dynamic regulation of labial tension [9] (Figure 1).

The larger bronchial half rings (A1 and A2) of males provide increased potential for transmitting force onto the labia and thus allow generation of a wider range of high fundamental frequencies. A thicker half ring is likely to be stiffer and therefore provides bending characteristics that enable fast recoil into the original position. The larger muscle mass of the male syrinx may be required to bend these stiffer half rings. As in other songbirds [27], muscle activity in the zebra finch is positively correlated with fundamental frequency (Goller, unpublished observations), supporting the hypothesis that increased tension is exerted on the labia in order to produce higher fundamental frequencies. Both ventral and dorsal syringeal muscles are simultaneously active during the production of zebra finch song syllables with the highest fundamental frequency [6], [29] (Goller, unpublished results), which can effect outward bending of the half rings, as pull is exerted on the ventral and dorsal ends.

The presence of a larger LL in females and the shape differences of the ML-MTM complex are not easily interpreted. Before neurotomy, the low frequency component of male distance calls and fundamental frequency in female distance calls is similar, while a high-frequency component can only be found in male distance calls [this study, 34,35]. After syringeal denervation fundamental frequency of calls is similar for males and females [29], [30], [31], [34], [36], [37], thus indicating that labial size is not an explanation for the observed differences. Because it is not clear which portion of the LL is actually vibrating during phonation, this morphological difference may not play a major role in the biomechanical control of sound frequency, but may be relevant in other aspects of sound production. The different design of the ML-MTM system may cause differences in the nonlinear stress response [38], which could affect the biomechanics of frequency control.

The relationship between driving pressure and fundamental frequency in the intact and denervated syrinx provides important insight into the relevance of the sexual dimorphism in frequency control. During phonation the interlabial gap of the syringeal valve is typically regulated by a combination of muscular activity [27] and passive movement driven by pressure differentials (Figure 1). In the denervated syrinx subsyringeal air sac pressure is higher during distance call production than before the denervation. It is possible that the pressure increase may be caused by a compensatory effort. The drop in fundamental frequency after neurotomy may trigger an increased effort to raise F0.

In the denervated syrinx, the driving air sac pressure alone regulates the opening of the syringeal valve. The relationship between fundamental frequency and subsyringeal driving pressure therefore depends on response characteristics of the labial tissue to stress and the interlabial opening at atmospheric pressure, i.e. in a neutral position of the syringeal valve. Interestingly, we found substantial differences in the pressure-frequency relationship between different individuals (Figure 4), indicating marked variation in either stress or valve lumen or both. However, the small sample size only allows the suggestion that there may be a systematic difference between males and females in these parameters.

The extent to which sound frequency can be changed by driving pressure is limited as indicated by the small slope of the linear regression for the denervated syrinx (Figure 4). Active control of labial tension is therefore the main mechanism that enables the production of a wide range of fundamental frequencies. Female zebra finches produce frequencies that are only slightly above the curve for pressure-driven frequency control, suggesting that muscular frequency control is limited. This conclusion again suggests that the above described biomechanical differences, combined with substantially increased muscle mass, are the main modifications that can account for the large difference in frequency range between male and female distance calls. Some song syllables contain even higher fundamental frequencies (up to 7 kHz) than are found in the distance calls [e.g. 3]. Frequency control of song syllables is also largely driven by muscular control of labial tension, as is illustrated by the large drop after denervation [29], [30], [31], [34], [36], [37]. The occurrence of high-frequency song components further supports specialization of the male syrinx for high frequencies, because only males sing in the zebra finch.

In summary, the enforced cartilage framework, a massive muscle package and much higher fundamental frequencies for a given range of subsyringeal air sac pressures in the intact, compared to the denervated, syrinx all suggest that the sexually dimorphic features play an important role in generating a wide fundamental frequency range, specifically by increasing this range toward higher frequencies in males. Sexually dimorphic vocal behavior therefore involves modification of the biomechanical system in addition to well-described differences in neural control [10], [11], [12] and syringeal muscles [13], [14], [15].

The fact that after denervation the solely pressure driven control of frequency only generates a much smaller frequency range confirms that control of labial tension through the neuromuscular system is an important aspect of achieving a broader range of acoustic features [27]. Much less control appears to be required for the production of the vocal repertoire of females. Interestingly, in a suboscine species, the kiskadee (*Pitangus sulfuratus*), bilateral denervation of the syrinx has a much less dramatic effect on the fundamental frequency of its song. Denervation of the syrinx leads only to minor frequency changes and does not cause a drastic change in the slope of the frequency-pressure relationship [39], unlike the results reported here for the male zebra finch. This apparent independence of direct muscular regulation of tension in the kiskadee therefore more closely matches frequency control in female zebra finches. The lack of muscular control of labial tension in a suboscine and the sexual dimorphism in this control aspect in the zebra finch strongly suggest that direct muscular control of frequency is an important step toward production of a broad frequency range in oscines. While we expect to find a similar strong muscular control of frequency in other male songbirds and in females that sing, we expect a more pressure-driven frequency control in suboscines.

The advantages and disadvantages of being able to produce higher frequencies are complex in all vertebrates [e.g. 40,41] because there are tradeoffs. Sound radiation from orifices and surfaces improves with increasing frequency [42], thereby improving radiation of high-frequencies from the beak compared to radiation of low frequencies for a given input. However, this apparent advantage is countered or paralleled by factors such as sound transmission in various habitats [43], sender and receiver perception [44], female preference [45], species recognition [46], and background noise [47]. It is safe to assume that the ability to generate a wide range of fundamental frequencies added an important degree of freedom that contributed to the success of oscine birds. The evolutionary step towards direct frequency control within birds parallels a similar step in mammals, which, unlike amphibians and reptiles, evolved a more direct muscular control of vocal fold tension [48], [49], [50].

## Methods

### Tissue collection and processing

We processed the syrinx of six male and six female zebra finches. The birds were deeply anaesthetized with a Ketamine/Xylazine combination (Sigma-Aldrich K-113; 2 µl/g body mass) and perfused intracardially with PBS followed by 5% neutral buffered formalin. The syrinx with short segments of trachea and the primary bronchi was carefully dissected out, and the isolated tissue was stored for 2 weeks in 10% Buffered Formalin Phosphate (Fisher Scientific; Fair Lawn, NJ, USA, cat. no. SF100-4) and for 8 hours in Decalcifier 1® (Surgipath Medical Industries, Inc., Richmond, IL, USA, cat. no. 00400) before further processing. The tissue was then embedded in paraffin and 5 µm cross sections of the complete syrinx were made. Data from males have been reported in part elsewhere [9].

### Staining

Adjacent sections were exposed to one of the following stains: hematoxylin and eosin (H&E) for a general histological evaluation; elastica van Gieson stain (EVG) to identify elastic fibers; trichrome stain (TRI) to demonstrate collagen fibers; alcian blue stain (AB) (pH 2.5) to determine mucopolysaccharides and glycosaminoglycans. We also performed a digestion procedure with 0.05 g bovine testicular hyaluronidase (Type I-S, Sigma-Aldrich, St. Louis, MO, USA; cat. no. H3506), solved in 100 ml buffer solution (94 ml Potassium phosphate, monobasic, 0.1 M, and 6 ml Sodium phosphate, dibasic, 0.1 M). Slides were first deparaffinized, then incubated in hyaluronidase at 37°C for 1 hour, and then washed in running water for 5 minutes and finally stained with alcian blue. The combination of the hyaluronidase digestion with a subsequent alcian blue stain increased specificity for various acid mucosubstances in the alcian blue stain. Alcian blue positivity is destroyed following prior incubation with hyaluronidase, if hyaluronan is a major component of the mucosubstances. All stains were performed in conjunction with control stains (artery for EVG; liver for TRI; umbilical cord for AB) on human tissue.

Micrographs were taken with a digital camera (AxioCam HRc, Carl Zeiss, Germany) combined with an Axioplan Zeiss microscope (Axioplan, Carl Zeiss, Germany) and computer software (Axiovision 40, v. 4.6.3.0, Carl Zeiss, Germany). Interpretation of the stains included a general description of labia, MTM, tympanum, bronchial half rings and intrinsic laryngeal muscles. Elastic fibers were randomly distributed and fiber content was overall very low. We therefore only present qualitative results.

To compare the intensity of the trichrome stains, images originally saved in RGB mode were converted to a 3-slice (red, green, blue) stack. The blue channel was further analyzed. The histogram function in Image J (version 1.41o; NIH open source) was used to determine the distribution of blue values in an image selection, intensity ranging from 0 (black) to 255 (white). The total pixel counts as well as the mean and modal blue value were compared between males and females. Staining with alcian blue was also quantified with the same procedure as described for the trichrome stains. The difference in the blue intensity before and after hyaluronidase treatment, was compared between males and females.

### Morphometry

Labium size measurements were based on cross-sectional area and cranio-caudal length for the medial labium (“ML”), on cross-sectional area for the lateral labium (“LL”) and on the length of the medial tympaniform membrane (MTM). The sizes of bronchial half rings (A1, A2, A3) and the pessulus (P) were estimated from cross-sectional area measurements. The size of the tympanum was based on its diameter about 3 mm below its cranial end.

Measurements were made with Image J. A curvilinear outline of the labia, the bronchial half ring and the pessulus was used to estimate cross-sectional area. A “segmented line” was drawn along the centerline of the medial labium starting at the pessulus and ending at the beginning of the MTM, and a second “segmented line” was drawn along the centerline of the MTM, to estimate the cranio-caudal length of ML and MTM. The area measurements (ML, LL; P, A1, A2, A3) and both length measurements (ML and MTM) were made at 10 points equally distributed over the dorso-ventral length of the labia, starting dorsally at the point where the pessulus and medial labium part from the dorso-lateral tracheal wall, and ending ventrally where pessulus and medial labium fuse again. All measurements were made in reference to a known distance measured at identical magnification.

Measurements (area, length) at 10 levels of female syringes were tested for differences along the dorso-ventral axis using a one-way analysis of variance (ANOVA). This test revealed significant differences along the dorso-ventral axis in the syrinx of males [9]. Measurements (area, length) of female syringes were also tested for differences between left and right syrinx using only the mid-organ section level (level 5) (two-sample t-test). This test revealed no significant differences for male syringes [9]. Differences between male and female syrinx were tested by two-sample t-test and are presented here.

To assess sexual dimorphism of the syrinx, we consider morphological differences of measured volumes between males and females that deviate from an expected difference based on isometric scaling with body size (i.e., volumes should be 1.16 fold greater in males than in females, because mean body mass of males was 1.16 fold greater than that of females in our sample of 6 individuals of each sex). If structures scale isometrcially with body mass, we expect a ratio of 1.04 for length measurements and 1.09 for area measurements. For example, the mass of the syrinx is sexually dimorphic as the male zebra finch syrinx is twice as heavy as that of females [14].

The CV for most parameters was relatively large and ranged from 11 to 44%. We therefore considered the mean CV of 25% as the confidence range around each expected isometric scaling as a cutoff value, i.e., the range of 0.78–1.3 for linear distances and 0.82–1.36 for area measurements.

### Nerve cut experiments

To assess how morphological differences may contribute to the frequency range of vocalizations, we recorded male and female distance calls before and after bilateral denervation of the syringeal muscles. The denervation enabled us to determine the respective contributions of lung pressure and muscle activity to frequency regulation (Figure 1A–E). After denervation, birds also produced sound during deep breathing (respiratory sounds) (Figure 2) which provided an estimate of the lowest fundamental frequency produced with minimal air sac pressure.

We denervated the syringeal muscles in order to determine the basic rate of oscillation of the labia. The oscillating frequency of labia depends on labia position (ad/abduction), their tension as well as subsyringeal air sac pressure. The first two functions are regulated by intrinsic syringeal muscles [6], [26], [27], [28], [29]. The third mechanism, i.e. fundamental frequency regulation by changes in the pressure differential between subsyringeal and atmospheric pressure, is less well understood. Disabling the intrinsic syringeal muscles will only leave the third mechanism in place, thereby creating a system regulated only by pressure changes, and allowing determination of vibration frequency of unstrained labia at the threshold pressure for phonation.

- Housing: Five adult males and five adult females were used in this study. All birds were more than 120 days old. Animals were separately housed in a small cage (32 cm×24 cm×25 cm).
- Neurotomy and pressure recording: The tracheosyringealis branch of the hypoglossal nerve runs laterally along the trachea. Using isoflurane anaesthesia, a skin incision was made in the ventral neck. On each side, a 1 cm-long piece of the tracheosyringealis nerve was excised in all ten birds.

In 3 of 5 birds of each sex, we also measured subsyringeal air sac pressure in the posterior thoracic air sac. An elastic belt with a Velcro tab on the back was placed around the thorax of each experimental bird. Birds were deprived of food and water for 1 h before surgery. Using isoflurane anaesthetic, a small hole was made in the abdominal wall into the left posterior thoracic air sac, and the tip of a flexible cannula (Silastic tubing; 1.65 mm o.d., 6 cm length) was inserted into this hole. The cannula was sutured to the rib cage. The cannula insertion site was sealed with tissue adhesive (Nexaband) to prevent leakage of air. The free end of the tube was connected to a piezoresistive pressure transducer (MSI, Model 1451), which was mounted on the Velcro tab.

A wireless custom-built transmitter-receiver system was used to transmit the transducer signal. The pressure transducer is DC excited and then connected to an amplifier. The amplified signal frequency modulates an oscillator connected to an antenna. The circuit board and components were then potted in epoxy for strength. The transmitter is very small and light weight and operates from a single Zinc Air cell for up to 30 days. It accepts pressure signals from 0 to 4 kPa and has a bandwidth of DC to 1 kHz. The transmitter antenna is capacitively coupled to positive (top of cage) and negative (bottom of cage) antennae. The difference between these antennae is amplified, filtered, and sent to a frequency to voltage converter reconstructing the original pressure signal from the transducer. A metal bird cage was used and grounded, providing shielding and noise immunity. The pressure transducer was calibrated before and after the experiments (Omega HHP-90). The signal is not affected by movements of the bird in the cage. The frequency modulated technology is immune to signal strength variations.

c) Sound recordings: Calls were recorded at three time points, 1) before the first surgery to implant the pressure transducer, 2) after the implantation of the pressure tube and before the nerve cut and 3) after the nerve cut. Calls and respiratory sounds were recorded with a microphone (AT8356; Audiotechnica, Stow, OH, USA) placed in front of the cage. The voltage output of the pressure transducer was recorded simultaneously with sound using a multi-channel A/D board (BNC 2110, National Instruments, Texas, USA) and Avisoft Recorder software (www.avisoft.de). All signals were recorded with a 44.1 kHz sampling frequency.
d) Call analysis: Zebra finches produce three call types [35]. Female calls display only little modulation, while male calls typically show modulation starting at a relatively high F0. We used distance and stack calls for analysis measuring the highest and the lowest fundamental frequency of male calls and the fundamental frequency in the middle of a call of the female calls (Figure 2). Differences in F0 before and after nerve cuts were tested within subjects with paired *t*-tests.

## Supporting Information

Supporting Information S1 Results: The histological composition of the labia as well as the dorso-ventral and left-right asymmetry of the syrinx comparing males and females.
(0.03 MB DOC)

Figure S1 Female zebra finch syrinx frontal sections (H&E stain) in ventral aspect of the organ. A: Schematic external ventral view of the excised organ in the upper part and of a frontal section of the syrinx (at mid-organ level indicated by the vertical plate in the upper part). The dotted square indicates the aspect that is shown in B through G. TL, tracheolateralis muscle; ST, sternotrachealis muscle; dTB and vTB, dorsal and ventral tracheobronchial muscle; dS and vS, dorsal and ventral syringeal muscle; ML, medial labium; LL, lateral labium; Tr, trachea; Br, primary bronchus; A1, A2, A3, first, second and third bronchial half ring. B through G: consecutive sections through the ventral aspect of the syrinx indicating how the bronchial half rings A2 (between D and E) and A1 (between F and G) become separately visible in the lateral wall of the bronchus and in the medial labium. Bars in B through G are 500 µm. H through K: The bronchial half rings A2 and A3 are composed of more than one type of material (cartilage/bone and hyaline cartilage). The images are larger magnifications of aspects indicated by squares in B, C and D. Bars in H through K are 100 µm.
(9.48 MB TIF)

Figure S2 Female zebra finch syrinx frontal sections (H&E stain). A: Section come from the ventral aspect of the syrinx, B: from mid-organ and C: from the dorsal syrinx. Pessulus (P), medial labium (ML), medial tympaniform membrane (MTM), lateral labium (LL), the first, second and third bronchial half ring (A1, A2, A3), interclavicular air sac (ICAS). Bars are 100 µm.
(4.55 MB TIF)

Figure S3 Masson's Trichrome stain of a zebra finch syrinx cross section. A: Section of a female syrinx showing the pessulus (P), medial labium (ML), medial tympaniform membrane (MTM) and first bronchial cartilage ring (B1). The square indicates the location of the higher magnification image in B and C. B: Part of the medial labium at larger magnification from a female syrinx. C: Part of the medial labium at larger magnification from a male syrinx. The arrows in B and C point to accumulations of collagen fibers (blue stain). Bars are 100 µm.
(3.44 MB TIF)

Figure S4 Elastica-van-Giesson stain of a zebra finch syrinx frontal section. A: Section of a female syrinx showing the pessulus (P), medial labium (ML), medial tympaniform membrane (MTM), lateral labium (LL), the second and third bronchial half ring (A2, A3), and the first bronchial ring (B1). The squares indicate the location of the higher magnification images in B to E. Arrows in B to E indicate longitudinal and cross sections of elastic fibers (black stain). Bars are 100 µm.
(10.08 MB TIF)

Figure S5 A female zebra finch syrinx frontal section stained with alcian blue before (A) and after Hyaluronidase digestion (B). Pessulus (P), medial labium (ML), medial tympaniform membrane (MTM), lateral labium (LL), the second and third bronchial half ring (A2, A3), bronchial ring (B). Bars are 100 µm.
(8.97 MB TIF)

Figure S6 A and B: Frontal section area measurements of medial (ML) and lateral labia (LL) in female syringes. Squares (left syrinx) and triangles (right syrinx) indicate means (error bars are standard deviation). C and D: Cranio-caudal length of the medial labium (ML) and the medial tympaniform membrane (MTM). Squares (left syrinx) and diamonds (right syrinx) indicate means (error bars are standard deviation). Level refers to ten subsequent section levels along the dorso-ventral axis of the syrinx organ.
(8.05 MB TIF)
