# Supplementary material for: Sexual Dimorphism of the Zebra Finch Syrinx Indicates Adaptation for High Fundamental Frequencies in Males
Source: PLoS One. 2010 Jun 29;5(6):e11368. doi: 10.1371/journal.pone.0011368 (PMC2894075; doi:10.1371/journal.pone.0011368)
Supplement: Supporting Information S1 — Results: The histological composition of the labia as well as the dorso-ventral and left-right asymmetry of the syrinx comparing males and females. (0.03 MB DOC) [file pone.0011368.s001.doc]

**Supporting Information**

**Results**

1) Histology

Bronchial half rings are important supportive components of the syrinx. Rotation of the third bronchial half ring (A3) moves the lateral labium into the bronchial lumen (Figure 1). The first and second bronchial half rings (A1, A2) arch with their end points into the ventral and the dorsal aspect of the medial labium. Consecutive sections in the ventral aspect of the female syrinx are shown in Figure S1. As the section level progresses dorsally the lateral and medial aspect of, first the second bronchial half ring (A2) (Figure S1D and E) and then the first half ring (A1) (Figure S1F and G) become separately visible. The respective medial aspects are embedded in the connective tissue of the medial labia. We refer here to all the supporting elements of the syrinx as “cartilaginous” because the amount of calcification was not the focus of this study but is variable. Evident from Figure S1I to K, the second bronchial half ring (A2) is calcified laterally whereas the medial aspect situated in ML is composed of hyaline cartilage. A3 is located exclusively in the lateral wall of the bronchus and has also a mixed composition in the ventral part of the syrinx (Figure S1C and 1K) but not in mid-organ level (Figure S2). This structural differentiation is presumably important for the bending flexibility of the support elements used in tensioning the labia (Riede & Goller 2010).

Labia change in shape, size and composition from dorsal to ventral, even at the mid-organ level (Figure S2) where presumably most of the tissue oscillation occurs. The lateral labium (LL) is embedded between the second and third bronchial half rings. It consists of connective tissue and stratified squamous epithelium, and it protrudes into the bronchial lumen. The medial labium (ML) consists of two layers of epithelium. Stratified squamous epithelium (2 to 3 layers of cells) faces toward the bronchial lumen, and a simple squamous epithelium of the interclavicular air sac forms the border to the interclavicular air sac. The two epithelial layers sandwich the connective tissue. Embedded in the connective tissue is a cartilage. The ML transform caudally into the medial tympaniform membrane (MTM), which are characterized by simple squamous epithelia on both sides.

Like in males, sections through the dorsal aspect of the syrinx indicate that muscle fibers attach medially to the dorsal ends of the bronchial half rings. Sections through the ventral aspect of the syrinx show that the ventral syringeal muscle attaches directly to the ML (Figures S1 and S2A).

a) Collagen

Collagen fibers were found in ML and LL (Figure S3A). Fibers are evenly distributed, the blue stain is mostly very faint (Figure S3 B,C). In the mid-organ sections differences in the intensity of blue stain in trichrome stains between males and females were not significant (two-sample t-test; N_1_ and N_2_ =6, T= -0.01, P=0.9).

b) Elastic fibers

Elastic fibers were found inconsistently in many areas of the ML and LL sections. Often none were present, sometimes they were concentrated in patches. High concentrations of elastic fibers were found in the ML near the bronchial half rings (Figure S4A). Positive elastic stain was also found in the transition region between the left and the right ML below the pessulus (Figure S4B). Most of the fibers showed a latero-lateral orientation from the left to the right ML. A third location with elastic fibers is the caudal end of the MTM (Figure S4C). Before the MTM attaches caudally to the first complete bronchial ring, connective tissue accumulates between the two epithelial layers and part of it are elastic fibers (igure S4D).

c) Hyaluronic acid

Alcian blue stains indicate the presence of mucopolysaccharides and glycosaminoglycans in the LL and ML (Figure S5A). The removal of hyaluronic acid by hyaluronidase digestion with subsequent alcian blue stain indicates that much of the positive stain in the labium can be attributed to hyaluronic acid (Figure S5B). Differences in intensity of blue stain before and after hyaluronidase treatment were significant (paired t-test; males: N=6, T= -3.3, P<0.01; females: N=6, T= -10.3, P<0.01). Differences between males and females in the intensity of blue stain were not significant (two-sample t-test; N_1_ and N_2_ =6, T= -0.22, P=0.82).

2) Histology/Morphology

a) Dorso-ventral and left-right variation in females

In females, the variation of the ML area at various section levels (Figure S6) is significantly different (F_1,120_ = 3.09, P < 0.01), and so is ML length (F_1,120_ = 2.7, P < 0.01), and MTM length (F_1,120_ = 5.1, P < 0.001), but not LL area (F_1,120_ = 0.88, P = 0.54). This indicates that ML shape in the female syrinx changes in the dorso-ventral axis (Supplement-figure 6), as it does in males (Riede, Goller 2010).

In females, the variation of all seven measures (ML-area, LL-area, ML-length, MTM-length, areas of A1, A2, A3, P) between the left and the right syrinx is not significantly different if all ten section levels are pooled for each individual (F_1,120_ = 0.3 – 1.3; P > 0.05), or if only one section level per individual is used (level 5: F_1,120_ = 0.04 – 3.2; P > 0.05).
