# Supplementary figures and images for: Sexual Dimorphism of the Zebra Finch Syrinx Indicates Adaptation for High Fundamental Frequencies in Males
Source: PLoS One. 2010 Jun 29;5(6):e11368. doi: 10.1371/journal.pone.0011368 (PMC2894075; doi:10.1371/journal.pone.0011368)

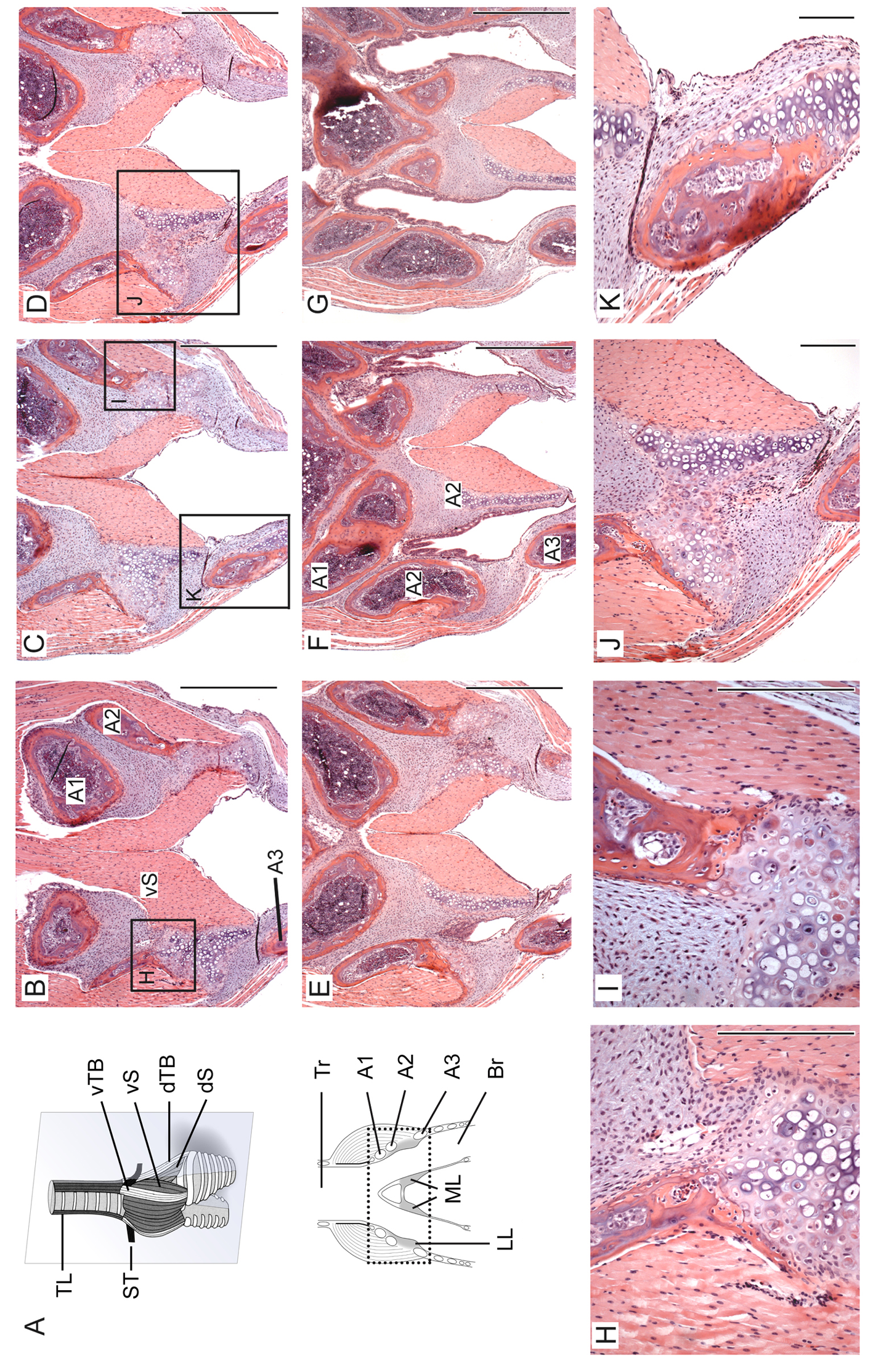

Supplement: Figure S1 — Female zebra finch syrinx frontal sections (H&E stain) in ventral aspect of the organ. A: Schematic external ventral view of the excised organ in the upper part and of a frontal section of the syrinx (at mid-organ level indicated by the vertical plate in the upper part). The dotted square indicates the aspect that is shown in B through G. TL, tracheolateralis muscle; ST, sternotrachealis muscle; dTB and vTB, dorsal and ventral tracheobronchial muscle; dS and vS, dorsal and ventral syringeal muscle; ML, medial labium; LL, lateral labium; Tr, trachea; Br, primary bronchus; A1, A2, A3, first, second and third bronchial half ring. B through G: consecutive sections through the ventral aspect of the syrinx indicating how the bronchial half rings A2 (between D and E) and A1 (between F and G) become separately visible in the lateral wall of the bronchus and in the medial labium. Bars in B through G are 500 µm. H through K: The bronchial half rings A2 and A3 are composed of more than one type of material (cartilage/bone and hyaline cartilage). The images are larger magnifications of aspects indicated by squares in B, C and D. Bars in H through K are 100 µm. (9.48 MB TIF) [file pone.0011368.s002.tif]

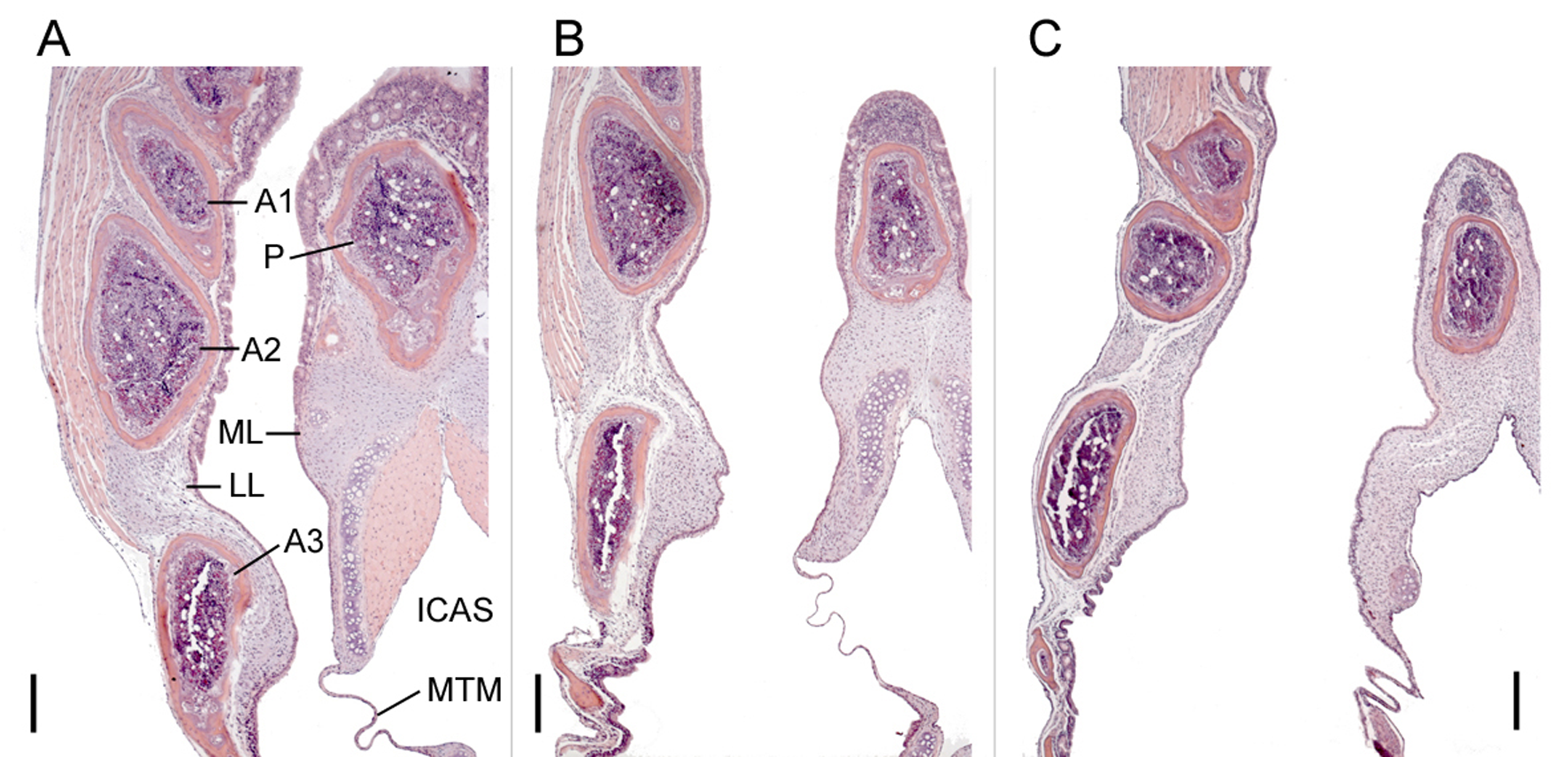

Supplement: Figure S2 — Female zebra finch syrinx frontal sections (H&E stain). A: Section come from the ventral aspect of the syrinx, B: from mid-organ and C: from the dorsal syrinx. Pessulus (P), medial labium (ML), medial tympaniform membrane (MTM), lateral labium (LL), the first, second and third bronchial half ring (A1, A2, A3), interclavicular air sac (ICAS). Bars are 100 µm. (4.55 MB TIF) [file pone.0011368.s003.tif]

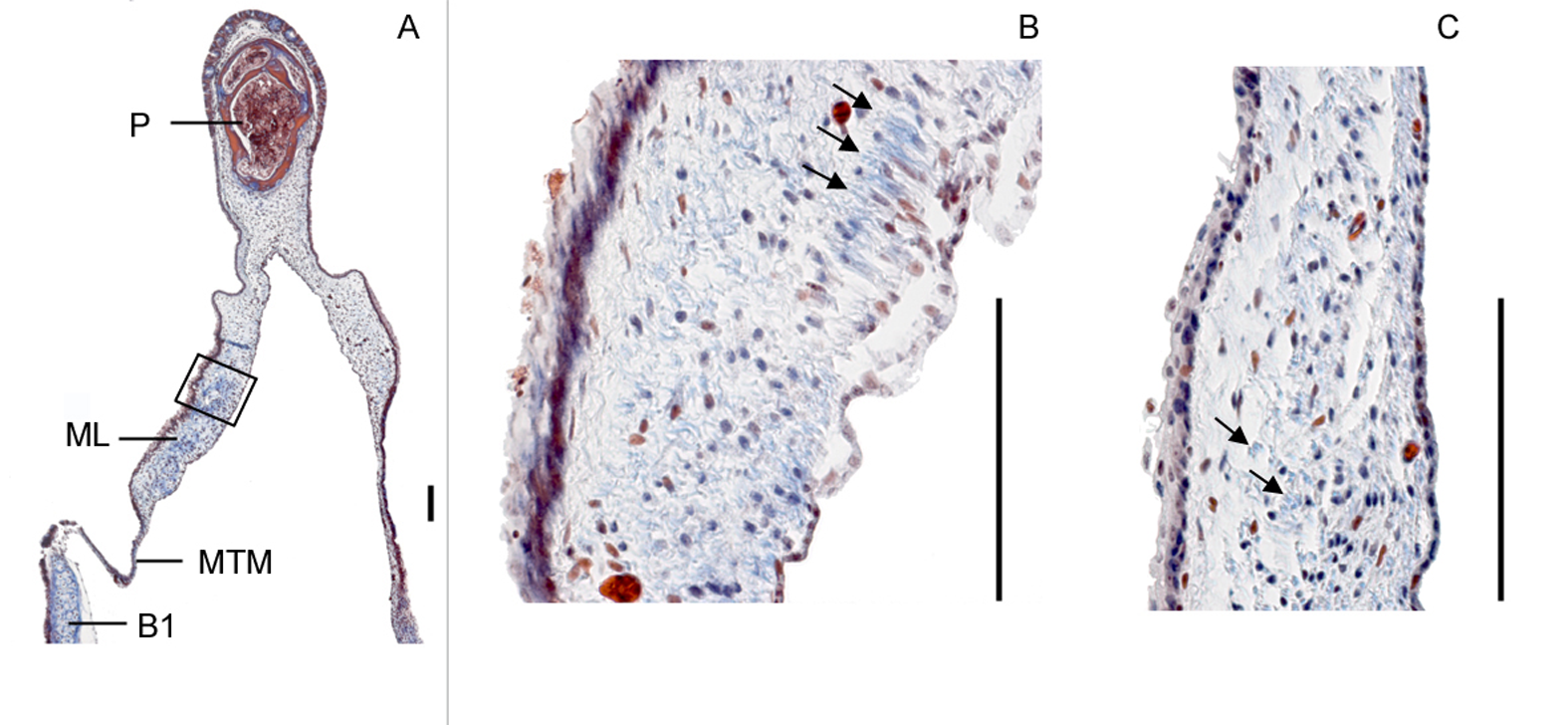

Supplement: Figure S3 — Masson's Trichrome stain of a zebra finch syrinx cross section. A: Section of a female syrinx showing the pessulus (P), medial labium (ML), medial tympaniform membrane (MTM) and first bronchial cartilage ring (B1). The square indicates the location of the higher magnification image in B and C. B: Part of the medial labium at larger magnification from a female syrinx. C: Part of the medial labium at larger magnification from a male syrinx. The arrows in B and C point to accumulations of collagen fibers (blue stain). Bars are 100 µm. (3.44 MB TIF) [file pone.0011368.s004.tif]

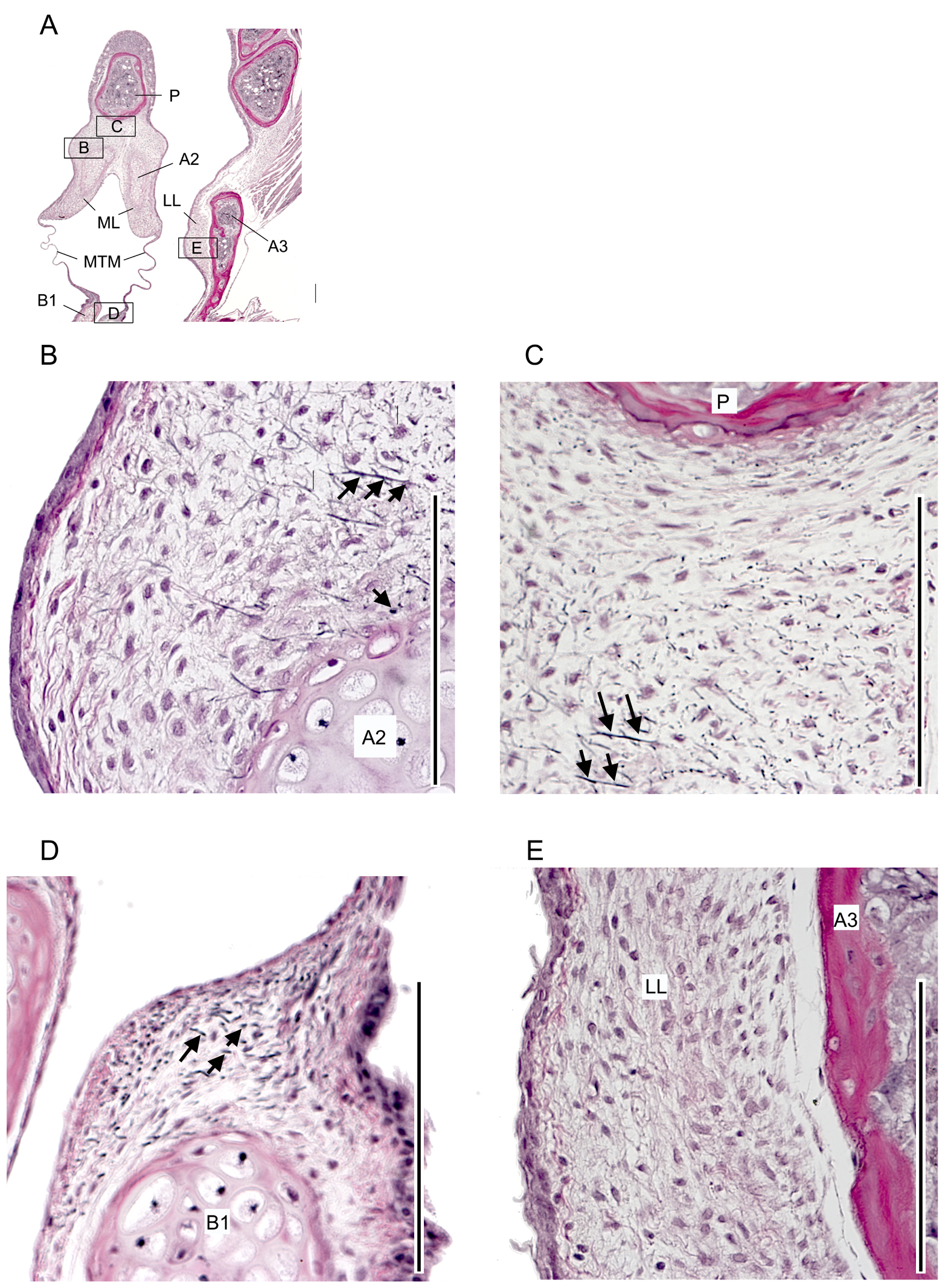

Supplement: Figure S4 — Elastica-van-Giesson stain of a zebra finch syrinx frontal section. A: Section of a female syrinx showing the pessulus (P), medial labium (ML), medial tympaniform membrane (MTM), lateral labium (LL), the second and third bronchial half ring (A2, A3), and the first bronchial ring (B1). The squares indicate the location of the higher magnification images in B to E. Arrows in B to E indicate longitudinal and cross sections of elastic fibers (black stain). Bars are 100 µm. (10.08 MB TIF) [file pone.0011368.s005.tif]

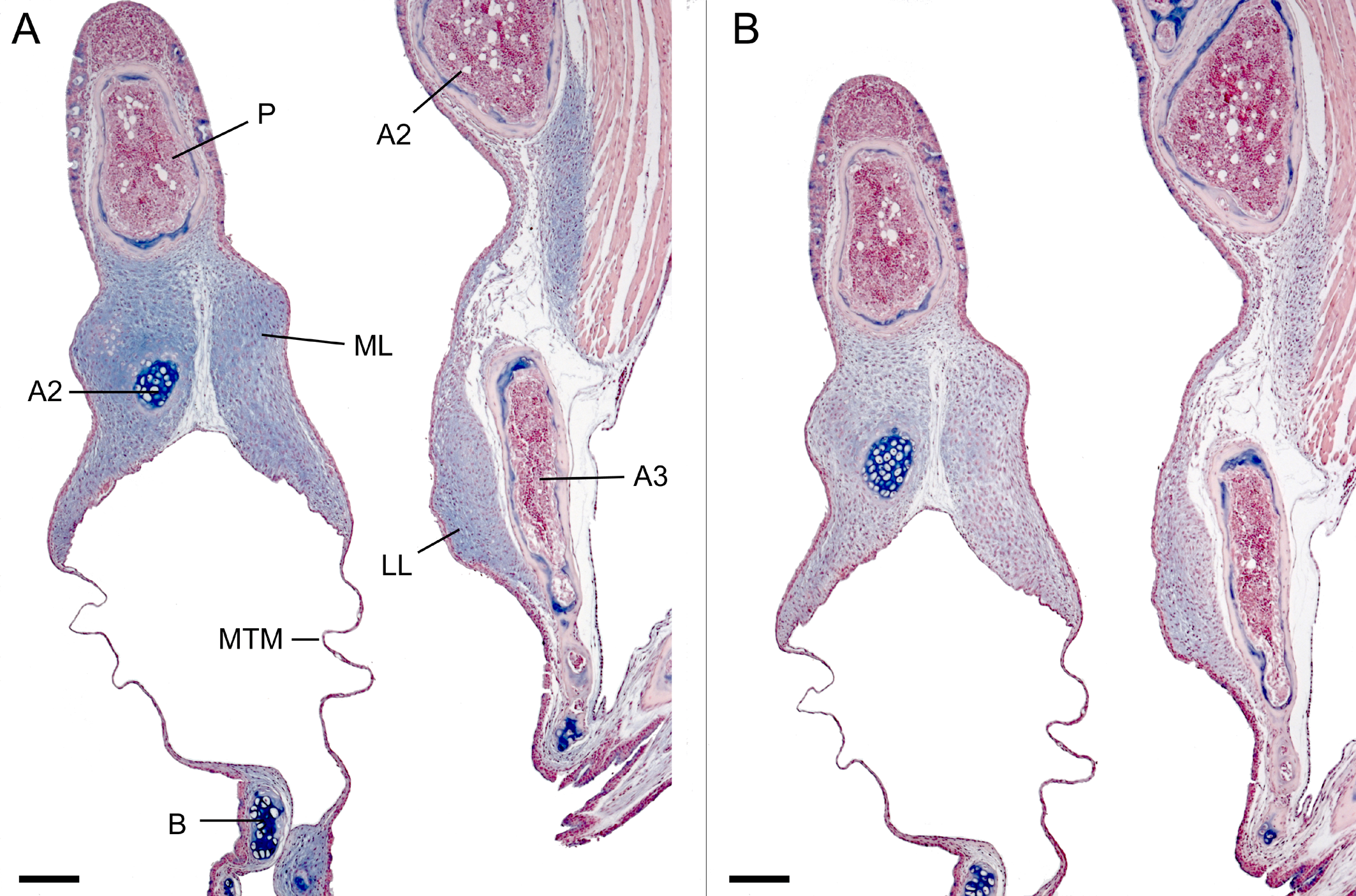

Supplement: Figure S5 — A female zebra finch syrinx frontal section stained with alcian blue before (A) and after Hyaluronidase digestion (B). Pessulus (P), medial labium (ML), medial tympaniform membrane (MTM), lateral labium (LL), the second and third bronchial half ring (A2, A3), bronchial ring (B). Bars are 100 µm. (8.97 MB TIF) [file pone.0011368.s006.tif]

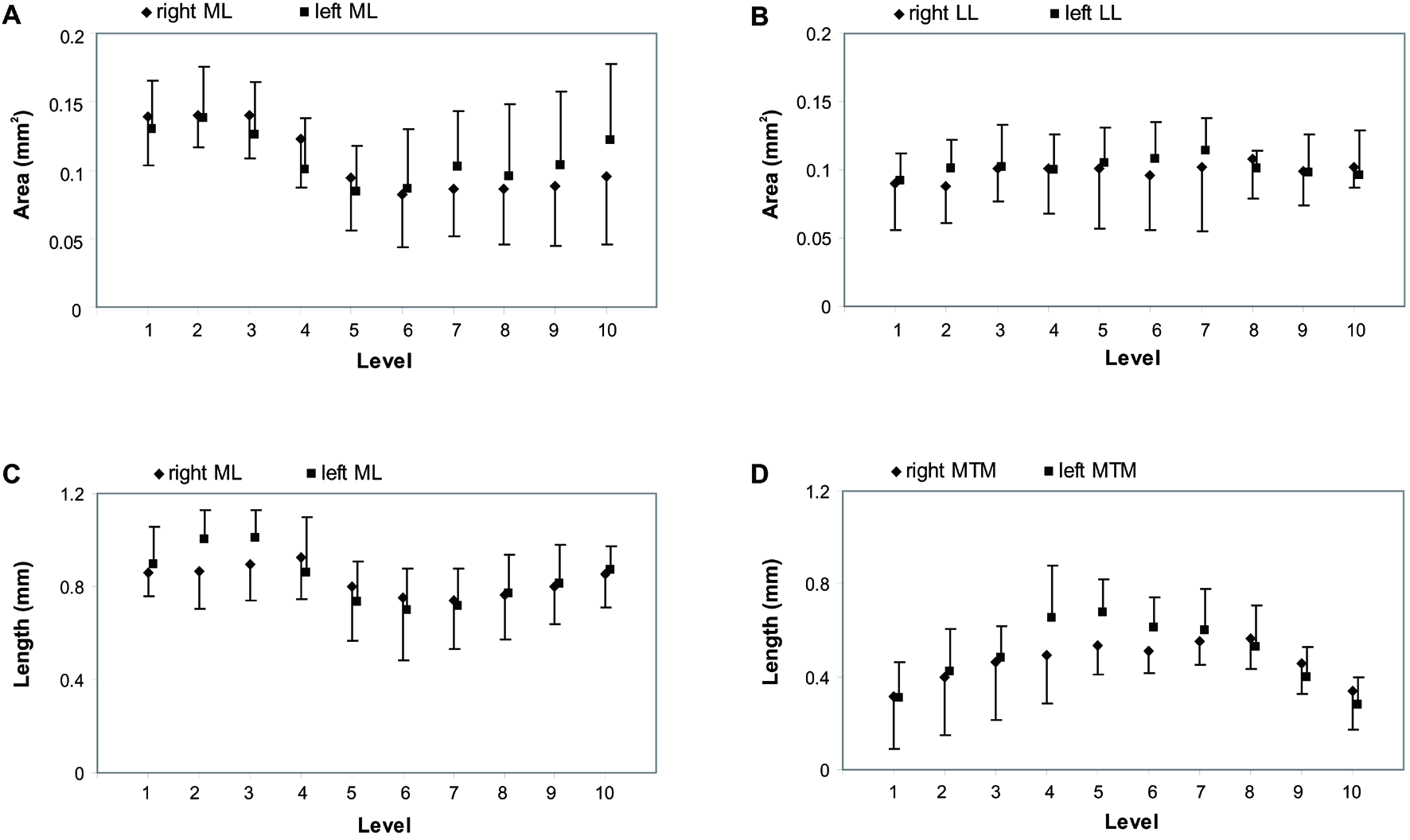

Supplement: Figure S6 — A and B: Frontal section area measurements of medial (ML) and lateral labia (LL) in female syringes. Squares (left syrinx) and triangles (right syrinx) indicate means (error bars are standard deviation). C and D: Cranio-caudal length of the medial labium (ML) and the medial tympaniform membrane (MTM). Squares (left syrinx) and diamonds (right syrinx) indicate means (error bars are standard deviation). Level refers to ten subsequent section levels along the dorso-ventral axis of the syrinx organ. (8.05 MB TIF) [file pone.0011368.s007.tif]
